# Social relevance drives viewing behavior independent of low-level salience in rhesus macaques

**DOI:** 10.3389/fnins.2014.00354

**Published:** 2014-11-05

**Authors:** James A. Solyst, Elizabeth A. Buffalo

**Affiliations:** ^1^Neuroscience Graduate Program, Emory UniversityAtlanta, GA, USA; ^2^Physiology and Biophysics, University of WashingtonSeattle, WA, USA; ^3^Yerkes National Primate Research CenterAtlanta, GA, USA; ^4^Washington National Primate Research Center, University of WashingtonSeattle, WA, USA; ^5^Center for Translational Social NeuroscienceAtlanta, GA, USA

**Keywords:** rhesus monkey, eye-tracking, face perception, scene perception, social cognition, memory, salience, attention

## Abstract

Quantifying attention to social stimuli during the viewing of complex social scenes with eye tracking has proven to be a sensitive method in the diagnosis of autism spectrum disorders years before average clinical diagnosis. Rhesus macaques provide an ideal model for understanding the mechanisms underlying social viewing behavior, but to date no comparable behavioral task has been developed for use in monkeys. Using a novel scene-viewing task, we monitored the gaze of three rhesus macaques while they freely viewed well-controlled composed social scenes and analyzed the time spent viewing objects and monkeys. In each of six behavioral sessions, monkeys viewed a set of 90 images (540 unique scenes) with each image presented twice. In two-thirds of the repeated scenes, either a monkey or an object was replaced with a novel item (manipulated scenes). When viewing a repeated scene, monkeys made longer fixations and shorter saccades, shifting from a rapid orienting to global scene contents to a more local analysis of fewer items. In addition to this repetition effect, in manipulated scenes, monkeys demonstrated robust memory by spending more time viewing the replaced items. By analyzing attention to specific scene content, we found that monkeys strongly preferred to view conspecifics and that this was not related to their salience in terms of low-level image features. A model-free analysis of viewing statistics found that monkeys that were viewed earlier and longer had direct gaze and redder sex skin around their face and rump, two important visual social cues. These data provide a quantification of viewing strategy, memory and social preferences in rhesus macaques viewing complex social scenes, and they provide an important baseline with which to compare to the effects of therapeutics aimed at enhancing social cognition.

## Introduction

For decades, eye tracking has been used to uncover how we explore the visual world and the features that guide our attention. Buswell was the first to explore this topic when he observed that fixations increased in duration over the course of viewing and speculated that image regions receiving many fixations of long duration were the “principal centers of interest” (Buswell, 1935). Subsequent formal analysis revealed that scene exploration begins with long saccades and quick fixations landing on highly informative regions as participants quickly orient to the global gist of the scene, with fixations then increasing in duration and saccades decreasing in amplitude as participants focus on local details (Antes, 1974).

This early work demonstrated that exploration of the visual world is a dynamic process that changes with experience and is driven by distinguishable features. The trace of this experience is retained not just within a given encounter but also across repeated episodes. When viewing repeated scenes, participants make fewer fixations and sample fewer regions compared to when the scene was novel, suggesting that participants retain knowledge of its contents (Smith et al., 2006). When presented with scenes that have been manipulated after the initial exposure, participants spend a greater amount of time investigating altered scene items than those repeated without manipulation, and this behavior correlates with the participant's explicit memory of the scene (Smith et al., 2006). Studies have also demonstrated that this viewing behavior depends on the integrity of medial temporal lobe structures. Amnesic patients with medial temporal lobe damage that includes damage to the hippocampus demonstrate impaired viewing behavior for manipulated scenes (Ryan et al., 2000; Smith et al., 2006; Smith and Squire, 2008).

In autistic individuals, eye tracking during free viewing of complex social scenes has revealed reduced attention toward the eyes and greater attention to the mouth compared to controls (Klin et al., 2002a; Jones et al., 2008; Jones and Klin, 2013). Functional imaging work has suggested that attention to the eye region of faces is linked to activation in the amygdala in autistic individuals (Dalton et al., 2005). Rhesus macaque monkeys provide an excellent model for understanding how single neurons contribute to attention to social stimuli, because exactly the same image viewing tasks can be used in humans and monkeys. Such tasks rely on natural gaze behavior, thereby reducing potentially confounding effects of extensive training upon task strategy, enhancing the face validity of the behavioral correlates investigated, and making direct comparisons to humans more valid. However, despite the high prevalence of disorders like autism that are characterized by impaired viewing behavior in social scenes, appropriate tasks for assessing these behaviors in rhesus macaques have not been as well explored.

Studies investigating social perception have almost exclusively used images of faces cropped from the body, finding that both rhesus macaques (Keating and Keating, 1982; Mendelson et al., 1982; Wilson and Goldman-Rakic, 1994; Guo et al., 2003, 2006; Gothard et al., 2004, 2009; Deaner et al., 2005; Ghazanfar et al., 2006; Nahm et al., 2008; Leonard et al., 2012) and humans (Haith et al., 1977; Walker-Smith et al., 1977; Janik et al., 1978; Althoff and Cohen, 1999; Henderson et al., 2005) prefer to view faces, particularly the eye region, compared to other stimuli. However, in natural settings, faces are rarely seen in isolation from bodies and other individuals and objects. Several groups have emphasized the importance of maintaining high ecological relevance when studying attention to social stimuli (Neisser, 1967; Kingstone et al., 2003; Smilek et al., 2006; Birmingham et al., 2008a,b, 2012; Riby and Hancock, 2008; Bindemann et al., 2009, 2010; Birmingham and Kingstone, 2009). While isolated faces direct attention to the face by design, faces embedded in complex scenes demand that the viewer select among many stimuli the ones that are most relevant. It has been suggested that this difference in stimulus complexity (Riby and Hancock, 2008) might explain why some studies have found that attention to faces is reduced in ASD (Klin et al., 2002b; Pelphrey et al., 2002; Trepagnier et al., 2002; Nacewicz et al., 2006; Spezio et al., 2007; Jones et al., 2008; Riby and Hancock, 2008; Sterling et al., 2008), while other studies reported no difference from neurotypical individuals (Van der Geest et al., 2002a,b; Bar-Haim et al., 2006; De Wit et al., 2008; Rutherford and Towns, 2008). A direct comparison of isolated faces and social scenes revealed that individuals with Asperger syndrome looked less at the eyes when faces were embedded in social scenes but were not different from neurotypicals when faces were presented in isolation (Hanley et al., 2012).

To our knowledge, only two studies have used social scenes when examining eye movements in monkeys (Berger et al., 2012; McFarland et al., 2013). McFarland and colleagues showed humans and male rhesus monkeys photos of either affiliative (grooming) or aggressive (chasing) interactions between two individuals from various primate species. They found that while both subject groups spent more time viewing faces compared to bodies, humans spent almost twice as much time viewing the individuals in the scene as did the rhesus. One important caveat is that the rhesus subjects used were not raised in a species-typical environment and spent only 3.1 s out of the available 10 exploring the images, of which only 8 images out of the 40 depicted conspecifics.

Apart from social revelance, some have suggested that attention to faces, particularly the eye region, is related to the high contrast between the eyes and the rest of the face (Ebitz and Platt, 2013; Ebitz et al., 2013). This hypothesis is motivated by the finding that during free viewing of natural scenes devoid of faces, attention is allocated to the most visually salient low-level features such as orientation contrast, intensity and color information (Itti and Koch, 2000; Parkhurst et al., 2002). However, the predictive power of visual salience has been challenged, citing the importance of the high-level “cognitive relevance” of items related to the needs and preferences of the viewer in determining which features are selected for attentive processing (Henderson et al., 2009). Supporting this view, visual salience does not account for fixations on objects of social relevance (faces and eyes) made by humans when viewing social scenes (Birmingham et al., 2009; Freeth et al., 2011; Levy et al., 2013), and adding information about features with high cognitive relevance (faces and text) to visual salience models dramatically improves their predictive power (Cerf et al., 2009). Here we aimed to assess the relative contributions of high-level cognitive relevance and low-level visual salience in the allocation of attention during social scene viewing, as well as the effect of experience on viewing behavior.

## Material and methods

### Data collection

Procedures were carried out in accordance with National Institutes of Health guidelines and were approved by the Emory University and University of Washington Institutional Animal Care and Use Committees. Three adult male rhesus monkeys (*Macaca mulatta*) were obtained from the breeding colony at the Yerkes National Primate Research Center Field Station where they were mother-reared in large, multi-family social groups for the first 3 years of life. Their weight and age at the start of the experiment was: M1: 19 kg, 9 years; M2: 19 kg, 10 years; M3: 13 kg, 11 years.

During testing, each monkey sat in a dimly illuminated room, 60 cm from a 19-inch CRT monitor, running at 120 Hz, non-interlaced refresh rate, with a resolution of 800 × 600 pixels. Eye movements were recorded using a noninvasive infrared eye-tracking system (ISCAN, Burlington, MA) that measured the position of the pupil and corneal reflection of the right eye. During testing, the subject's head was restrained with a head-holding post implanted under aseptic conditions. Eye movements were sampled at 200 Hz and saccades were detected offline using a velocity threshold of 30°/s and measured in degrees of visual angle (dva). Stimuli were presented using experimental control software (CORTEX, www.cortex.salk.edu). At the beginning of each behavioral session, the monkey was administered 2 mL of aerosolized saline solution intranasally through a Pari Baby™ pediatric mask placed over the nose (Pari Respiratory Equipment Inc., Midlothian, VA) using a Drive Pacifica Elite nebulizer (Drive Medical Design & Manufacturing, Port Washington, NY). Subjects were gradually acclimated to the nebulization procedure prior to the experiments using positive reinforcement and did not exhibit any signs of distress during saline administration at the time the experiments were conducted.

Following saline administration, the monkey performed an eye position calibration task, which involved holding a touch sensitive bar while fixating a small (0.3°) gray fixation point, presented on a dark background at one of 9 locations on the monitor. The monkey was trained to maintain fixation within a 3° window until the fixation point changed to an equiluminant yellow at a randomly-chosen time between 500 and 1100 ms after fixation onset. The monkey was required to release the touch-sensitive bar within 500 ms of the color change for delivery of food reward. During this task, the gain and offset of the oculomotor signals were adjusted so that the computer eye position matched targets that were a known distance from the central fixation point. Following the calibration task, the monkey performed either a delayed match-to-sample task or another calibration task identical to the 9-point task but with 63 locations covering the entire monitor in a grid with 4° spacing between each location. Data collected during the calibration task were used to compute a linear or polynomial transformation of the eye data to improve the calibration *post-hoc*.

**Figure 1:**
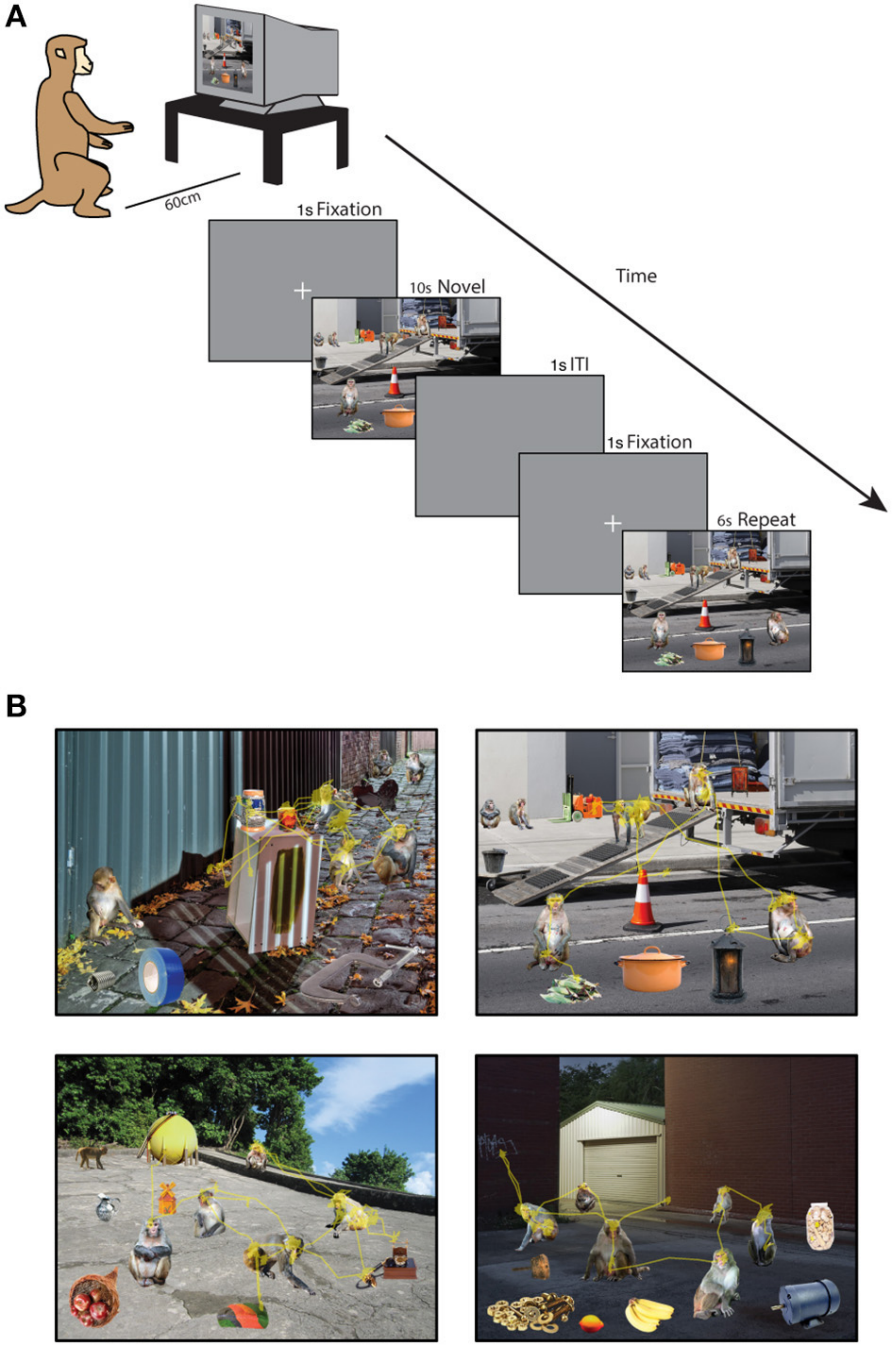
**Social scene viewing task. (A)** Three adult male rhesus macaques freely viewed images of social scenes composed of objects and unfamiliar rhesus monkeys while their point of gaze was monitored. In each session, 90 novel scenes were each presented twice for 10 s (Novel presentation) and 6 s (Repeat presentation) of cumulative viewing time. **(B)** Example scenes with the scan path overlaid showing the point of gaze during one trial.

Forty minutes after saline administration was completed, the monkey was tested on the Social Scene Viewing Task (Figure 1A), a variant of a scene memory task used to test memory in healthy and amnesic humans (Cohen et al., 1999; Ryan et al., 2000; Ryan and Cohen, 2004; Smith et al., 2006; Smith and Squire, 2008; Hannula et al., 2010; Chau et al., 2011). The monkey initiated each trial by fixating a white cross (the fixation target, 1°) at the center of the computer screen. After maintaining fixation on this target for 1 s, the target disappeared and a Novel picture of a social scene measuring 25° by 33° was presented (see *Scene Creation* for details about scenes). The image remained on the screen until the monkey accumulated 10 s of viewing time, and any fixations made outside of the image bounds were not counted toward this viewing requirement and were not analyzed. After a 1 s inter-trial interval, the monkey initiated a second presentation of the scene by fixating a white cross (1°) at the center of the screen for 1 s. The second presentation of the scene remained onscreen until the monkey accumulated 6 s of viewing time on the scene. The monkey was not rewarded during the scene presentation. Between each block of two scene presentations, the monkey was able to obtain reward by completing 3 trials of the 9-point calibration task. This procedure enabled us to maintain motivation and verify calibration throughout the session. In each session lasting approximately 50 min, 90 novel scenes were each presented twice for a total of 180 scene viewing trials.

### Scene creation

A total of 540 unique social scenes (6 sets of 90 scenes) were composed in Adobe Photoshop® by manually arranging cropped images of rhesus monkeys and objects (referred to collectively as items) onto a unique background scene (Figure 1B). The background scenes included mainly outdoor scenes and city streets, were relatively free of other objects, and were all of a similar spatial perspective. The objects were automatically cropped in Photoshop from stock photos (Hemera Technologies® Photo Objects 50,000 Volume 1) and included trucks, industrial equipment, furniture and fruit. To obtain source material for rhesus images, we used photos taken at the Yerkes National Primate Research Field Station in Lawrenceville, GA (courtesy of Dr. Lisa Parr) and the Caribbean Primate Research Center in Cayo Santiago, Puerto Rico (taken by James Solyst). From these images, we cropped 635 images of 307 rhesus macaques and 635 photos of objects in Photoshop. All of the monkeys had neutral facial expressions, and all of the items and backgrounds were novel to the subjects at the outset of the experiments.

Each monkey image was categorized according to gaze direction (direct or averted from subject), the visibility of the eyes (0, 1, or 2 eyes visible), age (infant & juvenile or adult), and sex (male, female, or undetermined). Gaze direction was considered direct if the eyes were directed at the camera and was otherwise considered averted. For monkeys in which the age and sex were unknown, these characteristics were assessed visually by two raters who made judgments using body size, facial morphology, genital appearance and distension of the nipples. Adults were discriminated from infants and juveniles by their larger body size, larger genitals in males, distended nipples in females and increased facial prognathism. Sex was discriminated by genital appearance, larger body size and wider facial structure in males and nipple distension in females. When sex could not be clearly determined (particularly in infants & juveniles), sex was coded as unknown and these images were not included in analyses of sex. Inter-rater reliability was measured using Cohen's κ and was very good for age (κ = 0.93) and all sex categories (Males:0.96, Females:0.96, Unknown:0.96).

After cropping the items, they were then automatically scaled to occupy one of three set areas (2, 1, or 0.4% of the scene) using custom JavaScripts that interfaced with Photoshop, ensuring that item size was precisely controlled. For each scene in a set of 90 scenes we used custom scripts in MATLAB® (The Mathworks, Inc.) to randomly select a novel background scene and a unique combination of items from the pool of rhesus macaques and objects. Each scene contained 6 objects and 6 monkeys of different identities, with 4 items scaled to each of the 3 potential sizes. In each scene, one of the two monkeys occupying 2% of the scene area gazed directly at the subject while all others had averted gaze. Within a set of 90 scenes, no item was repeated. Across the 6 sets of scenes, the same combination of items within a scene was never repeated, and no background scene was ever repeated. In order to minimize adaptation to specific individuals, images of a given monkey did not appear in the 5 subsequent scenes. To create a scene, items were added to the background scene as individual layers in Photoshop and manually arranged on the background to create a realistic perspective. No items were placed in the center of the scene to prevent incidental fixations after the center fixation cross was extinguished.

Each scene was randomly assigned to be either repeated without manipulation (Repeat, *N* = 30 scenes per session), or feature a replacement of a monkey (Replaced, Monkey, *N* = 30) or object (Replaced, Object, *N* = 30) in the second presentation. For Replaced Object scenes, an additional object was drawn with one randomly designated as the Replaced object and the other the Replacement object. For Replaced Monkey scenes, two juvenile or adult monkeys with two eyes visible were selected to be the Replaced and the Replacement. Infants were not used as Replaced or Replacement monkeys because of the difference between other monkeys in expected size. Repeat scenes selected one monkey with two eyes visible and one object to be compared to the replaced monkey or object in Replaced scenes. All items used in these comparisons were of the same size (1% of image area).

### Data analysis

Eye movements with a velocity above 30° of visual angle (dva) per second were classified as saccades, while all other eye movements were classified as fixations. Only fixations lasting longer than 60 ms were analyzed. Saccades originating from fixations outside of the screen were not included in the analysis of saccade amplitude. To analyze the location of fixations, regions of interest (ROIs) were created in Photoshop around the whole item for monkeys and objects, the background (whole image minus all items) and around the face and rump of monkeys. The face ROIs included the entire head and the rump ROIs included the monkey's posterior. Face and rump ROIs were manually drawn in Photoshop for each of 635 monkey images and then automatically scaled with the whole item to match each of the 3 potential scene item sizes. Whole item ROIs were created for each item using JavaScript to select an item's layer in the Photoshop scene and then expand the item's contours by 5 pixels (0.19 dva) to account for error in the accuracy of the eye position. Face and rump ROIs were also expanded by 5 pixels to account for error in eye position determination. Fixations on regions of overlap between ROIs due to this expansion were not included in analysis. Black and white images of the ROI for each item in the scene were then imported into MATLAB where the pixel coordinates of the ROI were extracted and used to filter the eye data and calculate the area occupied by the ROI and statistics about its saliency and redness within the scene image.

Salience of the image was computed in MATLAB by summing feature maps for color, edge orientation, and intensity contrast over multiple spatial scales (Itti et al., 1998). The resulting salience map was normalized from 0 to 1, ranging from the least salient pixel to the most salient. This produced an 800 × 600 pixel saliency map, which was used to calculate the mean of saliency values for pixels within ROIs. We will use the term “salience” to refer to the visual salience of low-level image features (e.g., contrast, intensity, color opponency), not to be confused with the more general usage of “salience” to describe items with high-level cognitive relevance (e.g., social, incentive, or emotional salience) (Klin et al., 2002a; Averbeck, 2010; Kirchner et al., 2011; Shultz et al., 2011; Chevallier et al., 2012; Prehn et al., 2013).

To measure the redness of secondary sexual skin color of the monkeys in the scenes, we first converted the RGB color map of each scene image to a hue-saturation-value map using MATLAB. Then within each face and rump ROI, we calculated the total number of pixels with a red hue (hue value >0.9), and for each of the 635 monkey images, we calculated the mean number of red pixels in each ROI across every appearance of the monkey within a scene. To determine if this measure showed a correspondence with perceived redness of the sex skin on faces and rumps, we compared the mean number of red pixels in monkeys categorized as red by two raters experienced with rhesus macaques to those that were not categorized as red. Inter-rater reliability was very good for both faces (Cohen's κ = 0.83) and rumps (Cohen's κ = 0.81), and we found that the mean number of red pixels was significantly higher in both red faces, *t*_(633)_ = 3.65, *p* = 0.0003, *g* = 0.39, (Non-Red: *M* = 88.12 ± 3.52, Red: 122.26 ± 11.12) and rumps, *t*_(633)_ = 8.81, *p* < 0.0001, *g* = 0.88, (Non-Red: *M* = 85.97 ± 4.03, Red: 179.59 ± 13.85) compared to the rest of the image pool. We took these results as a proof of concept that our method of quantifying redness of the monkey images corresponded to what human observers perceived as red secondary sexual color in rhesus macaques.

To quantify the eye movements, we measured fixation duration (average duration of a fixation), saccade amplitude (distance between fixations), the number of fixations, time spent viewing, latency to first fixation (time elapsed from beginning of trial to the initiation of the first fixation on an ROI), and the latency to revisit an item (time elapsed since the end of the previous fixation on the ROI and the beginning of the next transition into the ROI). The eye movement measures were averaged across all applicable ROIs within a scene presentation (e.g., all fixations that landed on monkeys) and were then averaged across all trials within each session. All estimates of error are expressed as standard error of the mean across sessions. The data were analyzed using independent-samples *t*-tests or ANOVAs from data pooled across all sessions from the 3 subjects, and significant group tests were followed up with tests of the data from each subject separately, reporting the proportion of subjects that demonstrated a significant result. Significant main effects were followed up with *post-hoc* comparisons using independent samples *t*-tests that were corrected for multiple comparisons using a false discovery rate (FDR) correction of *p*-values. Effect sizes for *post-hoc t*-tests were calculated in terms of Hedges' *g* (Hedges, 1981) ([mean_group1_ − mean_group2_]/pooled standard deviation) using the Measures of Effect Size Toolbox for MATLAB (Hentschke and Stüttgen, 2011). To analyze viewing behavior across time, we used a cluster-based, non-parametric permutation test to compare viewing behavior at separate time-points throughout the trial, correcting for multiple comparisons (Maris and Oostenveld, 2007).

Six sessions of 90 scenes (540 unique scenes), each scene presented twice, were administered for each monkey. Likely due to a strong preference for novel stimuli, subjects sometimes looked away from repeated images. To limit our analysis to trials where the subject was sufficiently engaged, we excluded a trial if greater than 1085 ms was spent looking outside of the image (95th percentile of all trials). Subjects varied significantly in the time they spent outside per trial, *F*_(2, 3233)_ = 121.45, *p* < 0.0001 (M1: *M* = 38.09 ± 16.79 ms, M2: *M* = 150.17 ± 16.79 ms, M3: *M* = 416.73 ± 20.99 ms). Subjects spent more time looking outside during the second presentation (P2) than the first (P1), *F*_(1, 3233)_ = 8.87, *p* = 0.0029 (P1: *M* = 171.13 ± 11.79 ms, P2: *M* = 232.18 ± 17.56 ms) and this novelty preference effect was stronger for M3, who spent the most time outside. Out of the 3240 trials collected, 175 in total were excluded based on time outside and the following proportion of all trials were excluded for each subject: M1:0.2, M2: 1, M3: 4%. An additional 19 trials were excluded from analysis due to errors in the display of the stimuli during the experiments, yielding a total of 3046 trials.

## Results

### Viewing strategy changes with experience

**Figure 2:**
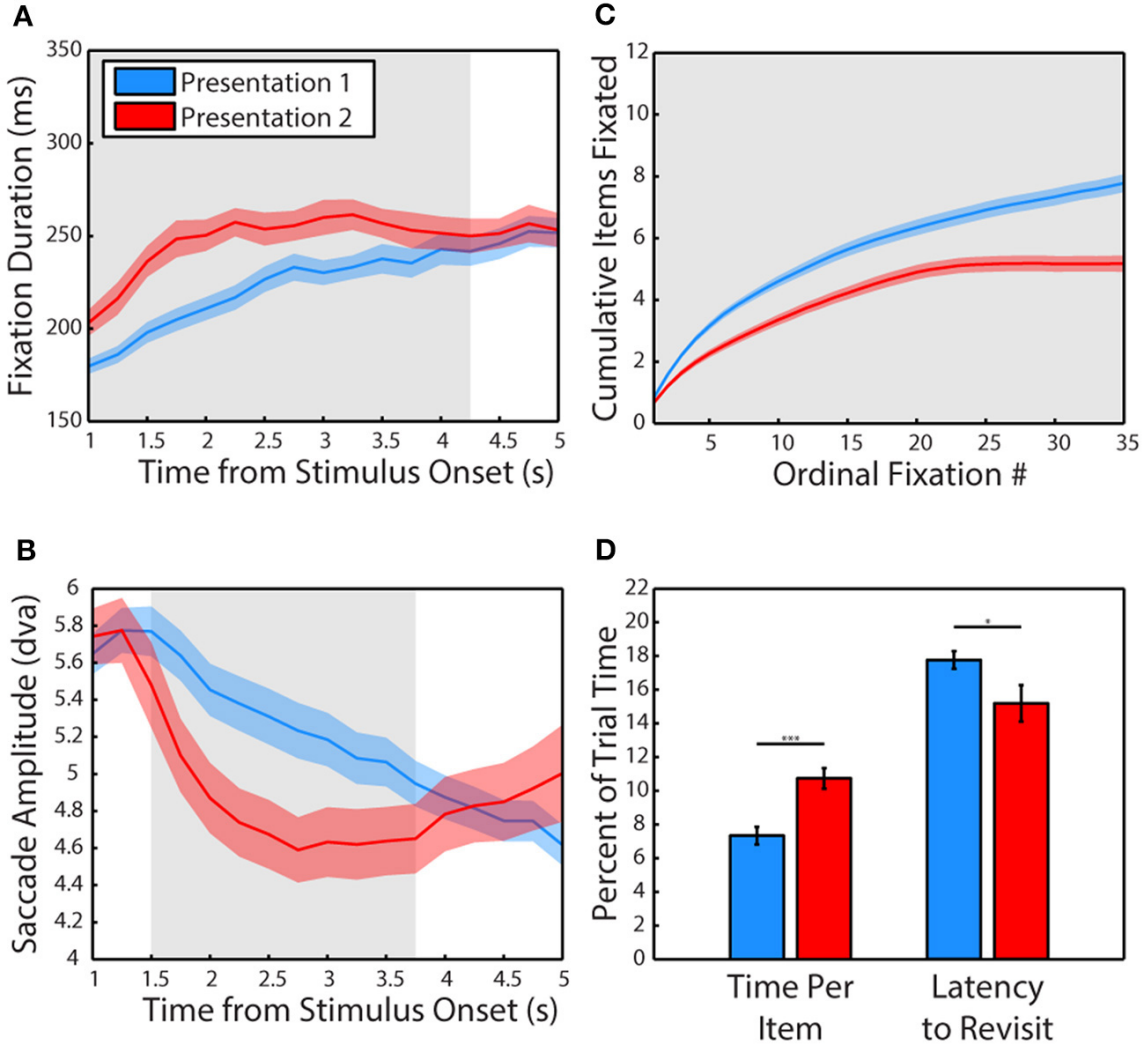
**Experience shifts viewing strategy from global to local. (A)** Mean duration of fixations across the first and second presentation of scenes. Data are plotted in 1 s bins stepped in 250 ms increments, with fixations included in a bin if the fixation was initiated during the time bin. Colored shading represents SEM across sessions and gray shading indicates periods of significant differences, calculated using a cluster-based non-parametric permutation test (*p* < 0.05, corrected for multiple comparisons, Maris and Oostenveld, 2007) for panels **(A–C)**. The second presentation lasted 6 s but only the first 5 s are plotted due to edge effects on fixation duration. **(B)** Amplitude of saccades across the first and second presentation of scenes. Same binning procedure as in A. **(C)** Cumulative items fixated (monkeys and objects combined) plotted across the first and second presentation by ordinal fixation number. **(D)** Time spent viewing each fixated item and latency to make a new transition into the item after an exit expressed in percent of trial time. Error is SEM across sessions. Asterisks represent significant differences (For all Figures: 1 star: *p* < 0.05, 2: *p* < 0.005, 3: *p* < 0.0005).

We first examined how viewing behavior changed from the first presentation of a scene (P1) to the second (P2). The data pooled from all 3 subjects revealed that fixations lasted significantly longer when viewing a scene for the second time (Figure 2A), *t*_(34)_ = 3.02, *p* = 0.005, *g* = 0.98, significant in 1/3 subjects, (P1: *M* = 202.72 ± 4.04 ms, P2: *M* = 223.23 ± 5.46 ms). A more sensitive, cluster-based, non-parametric permutation analysis (Maris and Oostenveld, 2007) of fixation duration across time (data binned in 1 s bins stepped in 250 ms increments) revealed that this effect was specific to the period of 0–4.25 s after stimulus onset when pooling data from all 3 subjects (significant in 2/3 subjects from 0 to 3.75 s).

Using this more sensitive time-resolved analysis method, saccades were found to be significantly smaller in amplitude during the second presentation from 1.25 to 3.75 s after stimulus onset when pooling data from all 3 subjects (Figure 2B, significant from 0 to 4.5 s in 2/3 subjects). However, a *t*-test of pooled data collapsed across the entire viewing period revealed that saccades were not significantly smaller during the second presentation (*p* > 0.1), although saccades were significantly smaller in 2/3 subjects. While these two subjects (M1& M2) showed robust decreases in saccade amplitude (Hedge's *g* of 1.91 and 1.24, respectively), subject M3 made significantly larger saccades during the second presentation (*g* = 1.87). The time-resolved analysis revealed that M3 made larger saccades at the end of the 2nd trial from 3 to 5 s, possibly related to the finding that this subject spent more time looking away from the scenes, particularly during the second presentation.

In the first 6 s of viewing, subjects viewed fewer items during the second presentation compared to the first (Figure 2C), *t*_(34)_ = 4.28, *p* = 0.0001, *g* = 1.4, significant in 3/3 subjects (P1: *M* = 6.67 ± 0.24 items, P2: *M* = 5.18 ± 0.25 items) and spent more time viewing each item, *t*_(34)_ = 4.23, *p* = 0.0002, significant in 3/3 subjects (P1: *M* = 7.33 ± 0.52% of trial time, P2: *M* = 10.73 ± 0.61% of trial time). Subjects were also quicker to revisit previously viewed items (Figure 2D), *t*_(34)_ = 2.14, *p* = 0.04, *g* = 0.7, significant in 2/3 subjects (P1: *M* = 17.75 ± 0.52% of trial time, P2: *M* = 15.18 ± 1.08% of trial time).

### Subjects remember scene contents

**Figure 3:**
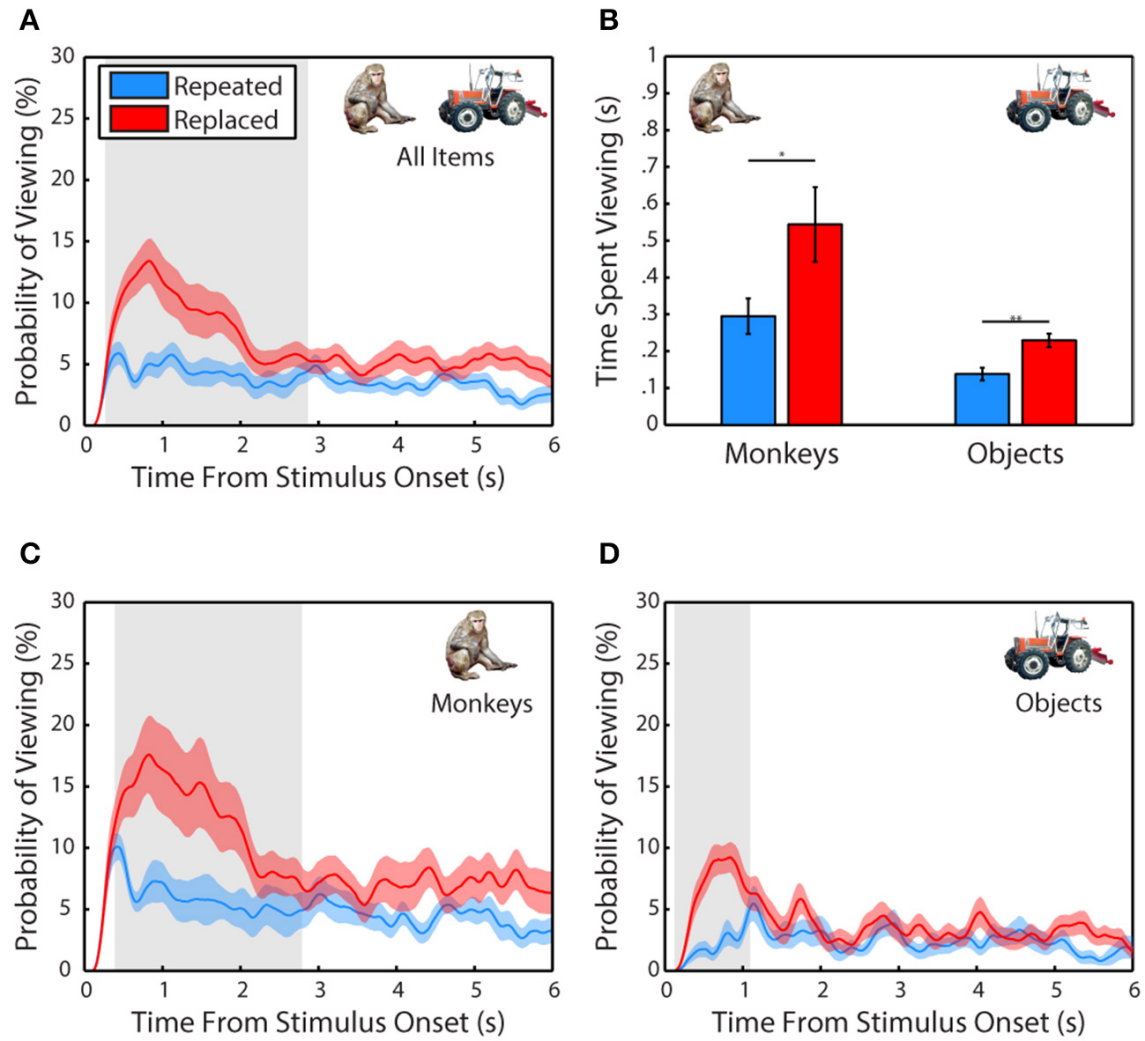
**Scene contents are remembered across experience. (A)** Probability of viewing items during the second presentation that were repeated without manipulation or replacements of an item from the first presentation. Only scenes where the repeated or replaced item was fixated during the first presentation were included. **(B)** Time spent viewing repeated and replacement monkeys and objects. **(C)** Same as in **(A)** but for monkeys only. **(D)** Same as in **(C)** but for objects only.

Next, we examined whether subjects demonstrated memory for scene items that were altered after the first presentation (Figure 3). A 2-way ANOVA pooled across each session from all 3 subjects included trial type (scene repeated without manipulation or featuring a replaced item) and item category (monkey or object) as factors and time spent fixating the repeated or replaced item in the second presentation as the dependent measure. This test revealed a significant main effect of trial type, *F*_(1, 71)_ = 8.78, *p* = 0.0001, significant in 3/3 subjects, with subjects spending more time viewing an item that was replaced than one repeated without manipulation, *t*_(70)_ = 2.66, *p* = 0.0128, *g* = 0.62, (Replaced: *M* = 386.59 ± 57.27 ms, Repeated: *M* = 216.38 ± 28.45 ms). We also found that there was a significant main effect of item category, *F*_(1, 71)_ = 16.86, *p* = 0.004, significant in 1/3 subjects, with subjects spending more time viewing a monkey than an object, *t*_(70)_ = 3.87, *p* = 0.0019, *g* = 0.9, (Monkey: *M* = 419.43 ± 59.11 ms, Object: *M* = 183.55 ± 14.66 ms). There was no significant interaction between item category and presentation, *F*_(1, 71)_ = 0.35, *p* = 0.55.

### Salience does not account for social viewing preference

**Figure 4:**
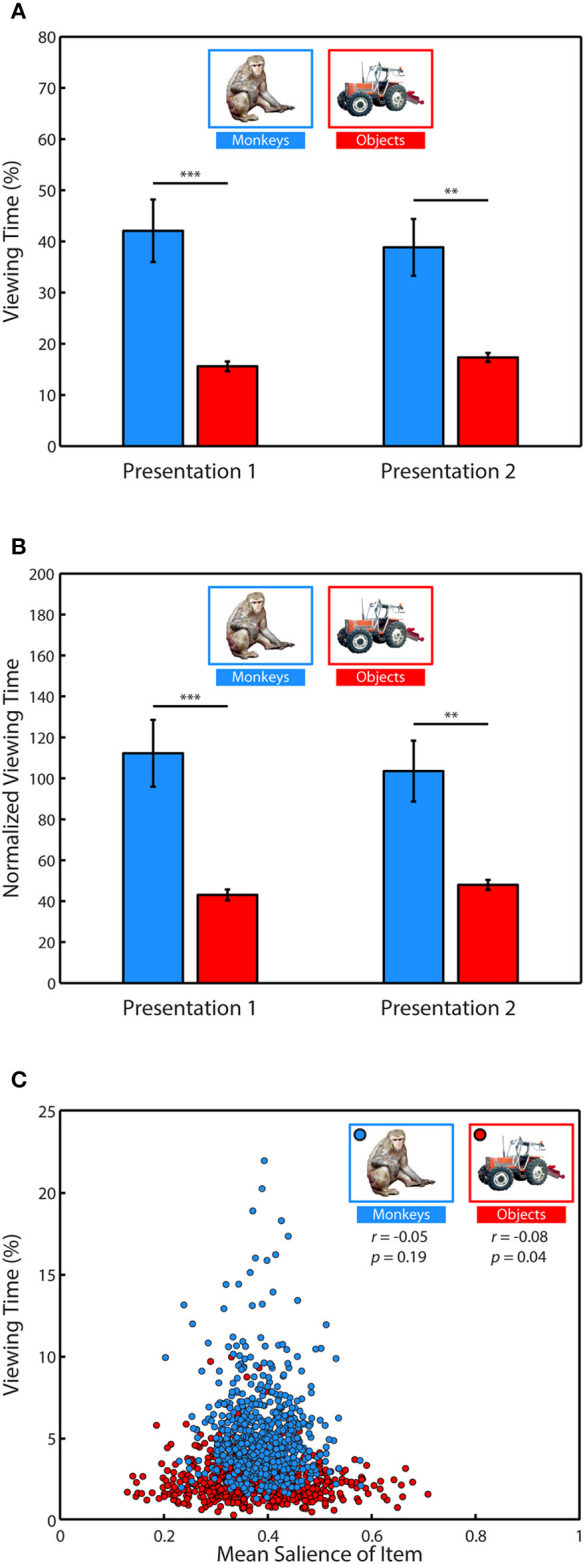
**Salience does not account for social viewing preference. (A)** Percent of fixation time spent viewing monkeys or objects. **(B)** Percent of fixation time divided by the mean salience of all pixels occupied by category items. **(C)** Correlation between the average percent of fixation time spent looking at each of the different monkeys and objects when they appeared in novel scenes and the average salience of those items.

To determine what subjects preferred to view when exploring the scenes, we performed a 2-way ANOVA with item category (monkeys or objects) and presentation number (first or second) as factors and the percent of fixation time spent looking at the monkeys and objects as the dependent variable. This analysis revealed a strong effect of category, *F*_(1, 71)_ = 32.91, *p* < 0.0001, significant in 3/3 subjects (Figure 4A), with monkeys being viewed more than objects, *t*_(70)_ = 5.80, *p* < 0.0001, *g* = 1.353, (Monkeys: *M* = 40.46 ± 4.08% of fixation time, Objects: *M* = 16.48 ± 0.65%). There was no significant effect of presentation on time spent viewing, *F*_(1, 71)_ = 0.03, *p* = 0.87, and no interaction between category and presentation, *F*_(1, 71)_ = 0.35, *p* = 0.55.

Next, we determined whether salience accounted for the preference for viewing monkeys, by first measuring whether image categories differed in salience, and whether subjects fixated more salient locations relative to the mean salience of the area (Table 1). An independent-samples *t*-test compared the mean salience (salience ranging from 0 to 1) of pixels occupied by monkeys and objects, and found that monkeys were slightly, but significantly more salient than objects, *t*_(6838)_ = 6.26, *p* < 0.0001, *g* = 0.15, (Monkeys: *M* = 0.3911 ± 0.0016 Objects: *M* = 0.3750 ± 0.0020).

**Table 1:** **Salience of image regions and fixations within those regions**.

	**Monkeys**	**Objects**
Mean Salience of ROI	0.3911 ± 0.0016	0.3750 ± 0.0020
Saliency at Fixation Location	0.3990 ± 0.0026	0.3758 ± 0.0026
Difference from mean Salience at Fixated Locations	0.0131 ± 0.0018	0.0066 ± 0.0016

*Salience of the image was computed in MATLAB by summing feature maps for color, edge orientation, and intensity contrast over multiple spatial scales. The resulting salience map was normalized from 0 to 1, ranging from the least salient pixel to the most salient*.

A 2-way ANOVA with item category (monkeys or objects) and presentation number as factors, and salience at fixation location as the dependent variable revealed a main effect of item category, *F*_(1, 71)_ = 41.15, *p* < 0.0001 (significant in 3/3 subjects), and a *post-hoc* comparison showed that the salience of fixations on monkeys was greater than objects, *t*_(70)_ = 6.4, *p* < 0.0001, *g* = 1.49, (Monkeys: *M* = 0.3990 ± 0.0026, Objects: *M* = 0.3758 ± 0.0026). There was neither a significant effect of presentation number, *F*_(1, 71)_ = 2.11, *p* = 0.15, nor a significant interaction between item category and presentation number, *F*_(1, 71)_ = 0.22, *p* = 0.64.

Next we asked whether subjects fixated the more salient regions of items, and if this differed by item category and presentation. We first performed a one-sample *t*-test of the hypothesis that the average difference between the mean salience of an item and the salience at fixation location in each session came from a distribution with a mean of zero (i.e., salience at fixated locations within an item was no different than the mean salience of the item). This test showed that subjects fixated locations within items that were more salient than the item's mean salience, *t*_(71)_ = 7.84, *p* < 0.0001, *g* = 0.91, *M* = 0.0098 ± 0.0013. To determine whether this differed by item category or presentation, we performed a 2-way ANOVA using the same dependent variable with item category and presentation as factors. This analysis revealed a significant main effect of item category, *F*_(1, 71)_ = 7.17, *p* = 0.009 (significant in 2/3 subjects), with subjects fixating relatively more salient parts of monkeys than objects, *t*_(70)_ = 2.68, *p* = 0.009, *g* = 0.62, (Monkeys: *M* = 0.0131 ± 0.0018, Objects: *M* = 0.0066 ± 0.0016). There was no significant main effect of presentation, *F*_(1, 71)_ = 1.81, *p* = 0.18, nor a significant interaction between item category and presentation, *F*_(1, 71)_ = 0.25, *p* = 0.62. The means and differences between mean salience and the salience at fixated regions are reported in Table 1.

Given these differences in salience between monkeys and objects, we reevaluated viewing preference in each trial by dividing the percent of fixation time spent viewing these categories by the mean salience of the region (Figure 4B). Using this normalized viewing measure as the dependent variable, we performed a 2-way ANOVA with item category (monkeys or objects) and presentation number (first or second) as factors. Consistent with the previous analysis using data not normalized by salience, there was a significant main effect of item category, *F*_(1, 71)_ = 31.11, *p* < 0.0001, (significant in 2/3 monkeys, *p* = 0.0559 in the other) with monkeys being viewed more than objects, *t*_(70)_ = 5.64, *p* < 0.0001, *g* = 1.32, (Monkeys: *M* = 107.86 ± 10.89 normalized viewing time, Objects: *M* = 45.5098 ± 1.822). There was no significant main effect of presentation number, *F*_(1, 71)_ = 0.03, *p* = 0.8631, and no significant interaction between item category and presentation, *F*_(1, 71)_ = 0.37, *p* = 0.5439.

To further examine whether time spent viewing an item was related to saliency, we next asked whether specific items with higher salience were viewed more than items with lower salience. To address this we calculated the mean percent of fixation time that was spent looking at each of the 635 different monkey and object images when they appeared throughout the scenes and correlated this value with the mean salience of those images as they appeared in the scenes. We found no significant correlation between the salience of a monkey and time spent viewing it (Pearson's linear correlation coefficient, *r* = −0.05, *p* = 0.19), and a weak but significant relationship for objects (*r* = −0.08, *p* = 0.04), such that objects viewed longer tended to be less salient (Figure 4C). Together, these results demonstrate that subjects preferred to view objects of social relevance and that salience did not account for this preference.

### Social relevance drives viewing behavior

**Figure 5:**
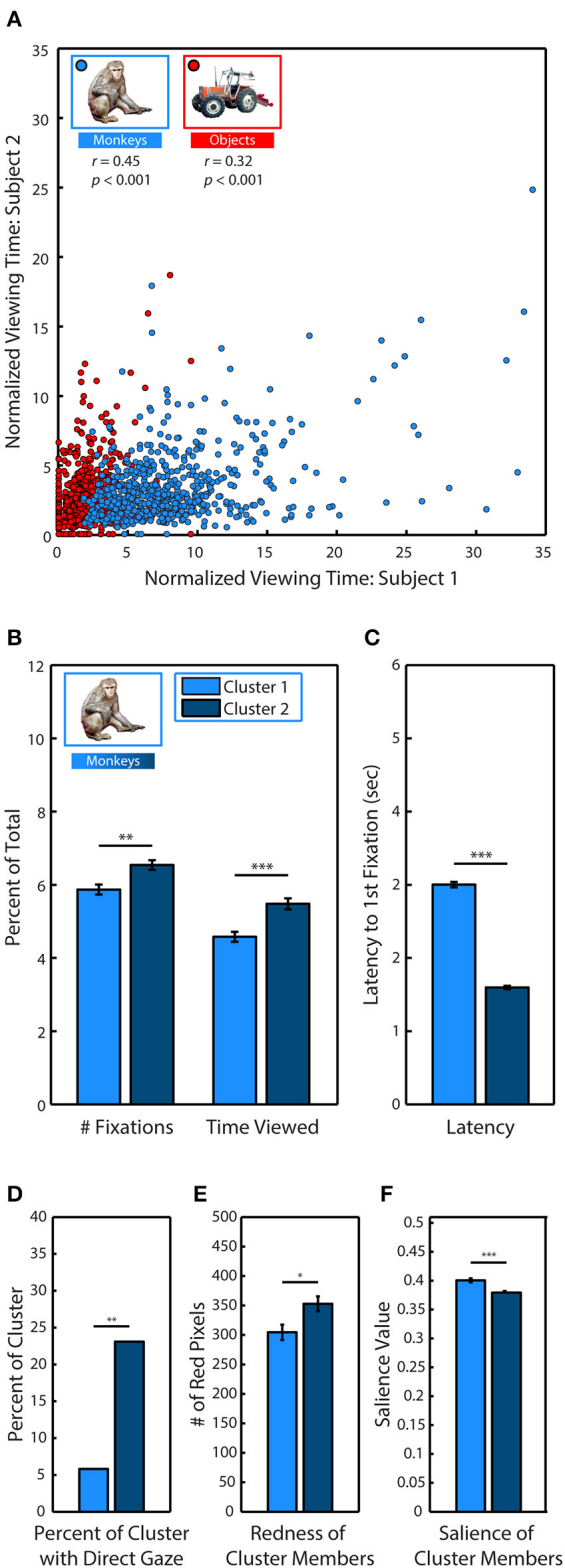
**Social relevance drives viewing behavior. (A)** Pearson's linear correlation between subjects M1 & M2 in the average percent of trial time spent looking at each of the different monkeys and objects when they were fixated in novel scenes. Because items differed in size, viewing time was divided by the percent of the image occupied by the item. **(B)** *k*-means clustering analysis of viewing statistics during the first presentation for each of the 635 different monkeys revealed two distinct clusters. Members of Cluster 2 (C2) were fixated significantly longer, and with more fixations than members of Cluster 1 (C1). **(C)** Same as **(B)** but for latency to first fixation. **(D)** Percent of cluster members with direct gaze. **(E)** Mean number of red pixels in cluster members. **(F)** Mean salience of cluster members.

After identifying monkeys as a highly viewed stimulus category, we examined whether specific characteristics of individual monkeys could explain viewing behavior. For each subject, we first calculated the percent of trial time spent viewing specific monkeys and objects across every appearance in the scenes. We divided this looking time by the percent of the image occupied in order to account for varying size, and we then measured how correlated the subjects were in their preferences. Instances when monkeys and objects replaced an item from the first presentation were excluded from analysis to avoid any influence of memory. During the first presentation of a scene, pairs of subjects were strongly correlated (Figure 5A) in the time they spent viewing specific monkeys (Pearson's linear correlation coefficient, M1–M2: *r* = 0.45, M1–M3: *r* = 0.24, M2–M3: *r* = 0.33, all *p* < 0.0001), as well as objects (M1–M2: *r* = 0.32, M1–M3: *r* = 0.13, M2–M3: *r* = 0.24, all *p* < 0.0001). To determine whether subjects showed stronger similarity in their preferences for monkeys compared to objects, we compared the between-subject correlations for monkeys and objects using Fisher's *z* transformation. This analysis demonstrated that subjects were significantly more correlated in the time they spent viewing monkeys compared to objects (M1–M2: *z* = 2.55, M1–M3, *z* = 2.03, M2–M3, *z* = 1.75, all *p* < 0.05).

After discovering that subjects were strongly correlated in their preferences for specific monkeys, we next used *k*-means clustering analysis to determine if specific monkeys formed discriminable groups based on viewing statistics. We limited our analysis to the first presentation and took the average across all subjects because subjects showed strong correlations in their preferences during this period. For each of the 635 monkey images, we calculated the percent of total fixations that were made on the monkey, the percent of trial time spent fixating the monkey and the latency to fixate the monkey after the trial began. Instances when monkeys and objects replaced an item from the first presentation were excluded from analysis to avoid any influence of memory. Measures calculated as a percent of total (fixations & time viewed) were divided by the percent of the image occupied by the monkey. To determine if the data formed distinct clusters and, if so, identify the optimal number of clusters for the data, we conducted a silhouette analysis that measured the separability of clustered data points by plotting the mean distance between each data point (each monkey) for each cluster in the 3 dimensional data space (Rousseeuw, 1987; Gan et al., 2007). Taking the mean of these distances revealed that clustering the data into two clusters (C1 & C2) resulted in distinct clusters with the highest separation between clusters (2 clusters: *M* = 0.73; 3: *M* = 0.69; 4: *M* = 0.70; 5: *M* = 0.70).

Compared to C1 (*N* = 242), the monkeys in C2 (*N* = 393) were viewed earlier, *t*_(633)_ = 33.91, *p* < 0.0001, (C1: *M* = 2.99 ± 0.04 s, C2: *M* = 1.59 ± 0.02 s), longer, *t*_(633)_ = 4.10, *p* < 0.0001, (C1: *M* = 4.58 ± 0.14, C2: *M* = 5.48 ± 0.15) and with more fixations, *t*_(633)_ = 3.36, *p* < 0.0001 (C1: *M* = 5.87 ± 0.13, C2: *M* = 6.54 ± 0.13) (Figures 5B,C).

To determine the characteristics of the monkeys in C2 that were viewed earlier and longer, we compared the prevalence of different attributes between each cluster. Before the experiment began, each monkey image was categorized according to the visibility of the eyes (0, 1, or 2 eyes visible), age (infant & juvenile or adult), sex (male, female, or undetermined), and gaze direction (direct or averted from subject). A significantly greater proportion of monkeys in C2 had direct gaze, χ^2^_(17.49, 1)_, *p* < 0.0001, [C1: 21 out of 242 (8.68%), C2: 84 out of 393 (21.37%)] (Figure 5D). There were no significant differences between clusters in regards to visibility of the eyes, age or sex.

In male and female rhesus macaques, the redness of sex skin around the face and rump increases during the mating season (Baulu, 1976), and adult males and females spend more time looking at red faces and rumps (Waitt et al., 2006; Gerald et al., 2007). We compared the mean number of red pixels in category members in each cluster and found that monkeys in C2 (*M* = 304.56 ± 12.96 red pixels) were significantly redder than those in C1 (*M* = 352.96 ± 12.57), *t*_(633)_ = 2.55, *p* = 0.01 (Figure 5E).

Finally, we found that monkeys in C2 were significantly less salient than those in C1, *t*_(633)_ = 4.75, *p* < 0.0001, (C1: *M* = 0.393 ± 0.003, C2: *M* = 0.372 ± 0.003) (Figure 5F).

## Discussion

To date, experiments using social scenes have been limited by potentially confounding variability present in uncontrolled stimuli as well as the extensive time and effort required to draw regions of interest around scene items and analyze the resulting data. As a result, low numbers of stimuli have been used and scene content has been characterized at relatively superficial levels, if at all. Inspired by studies using composed scenes (Melcher and Kowler, 2001; Henderson and Hollingworth, 2003; Unema et al., 2005a; Underwood et al., 2006; Birmingham et al., 2008b), we developed a semi-automated system for constructing hundreds of novel scenes from an image library of background contexts, objects and rhesus monkeys. This novel method permits control and characterization of scene content, and opens up new avenues for investigating memory and the role of scene content through manipulation of scene items.

Using this approach, we found that subjects shifted their viewing strategy with experience and demonstrated memory for scene content. Consistent with previous reports in humans, during the initial viewing, monkeys made fixations that steadily increased in duration and saccades that steadily decreased in amplitude (Buswell, 1935; Antes, 1974; Irwin and Zelinsky, 2002; Melcher, 2006; Pannasch et al., 2008). Interestingly, when a scene was viewed a second time, this change occurred much more rapidly. Only 2 s after the beginning of the second viewing, fixation duration and saccade amplitude reached levels similar to what was observed 5 s into the first trial. This increase in fixation duration with repeated viewing is in agreement with findings of a “repetition effect” in humans in which fixation durations are longer when viewing previously viewed images, demonstrating memory for scene contents (Althoff and Cohen, 1999; Ryan et al., 2007).

Apart from this general effect on scene viewing, we also investigated how subjects viewed particular items and whether this changed upon repeated viewing. We found that compared to the first viewing, subjects fixated on average about 1.5 fewer of the total 12 items during the same time period, which is analogous to the sampling of fewer image regions (Ryan et al., 2000). This change was accompanied by an increase in the time spent viewing each fixated item, and a decrease in the latency to revisit previously viewed items. Together with the observed increase in fixation duration and decrease in saccade amplitude, these changes suggest a shift in viewing strategy from an orientation to scene contents at a global level to a more elaborative focus on local detail. This shift may reflect a narrowing of focus onto items of high interest, which is consistent with a recent study finding that locations that are fixated by a high proportion of human observers are also viewed with longer fixations and shorter saccades (Dorr et al., 2010). A distinction between global and local viewing strategy based on fixation duration and saccade amplitude has also been made for humans viewing complex scenes (Unema et al., 2005b; Pannasch et al., 2008; Tatler and Vincent, 2008), and our data now extend this finding to non-human primates.

We also found that when an item was replaced by a new item in the repeated viewing, it was viewed longer than one that was repeated without manipulation, replicating the relational memory effect observed in humans (Ryan et al., 2000; Smith et al., 2006). These data suggest that subjects remembered the contents of the scene across repeated encounters, confirming previous work showing that memory for scene items persists across time (Melcher, 2001, 2006; Melcher and Kowler, 2001).

Despite decades of eye movement research, the characteristics of scene contents that are viewed by humans and monkeys during free viewing remain poorly understood. One prominent theory argues that simple low-level features of an image determine fixation location, with these salient locations being viewed more than would be predicted by chance during free viewing (Parkhurst et al., 2002). However, this hypothesis does not account for the existing priors and preferences of an organism that are developed over many interactions with its environment as it searches for food and mates. Encapsulating this alternative viewpoint is the cognitive relevance hypothesis, a theory which proposes that visual features are given specific weights based on the needs of the organism (Henderson et al., 2009). Indeed, objects in scenes are better predictors of fixation location than saliency, and the saliency of objects contributes little extra information despite the finding that memorable objects are often highly salient (Einhäuser et al., 2008). Perhaps one of the most important object categories for any organism, and especially group-living primates, are conspecifics.

Rhesus monkeys find social stimuli highly rewarding (Butler, 1954; Humphrey, 1974) and will even sacrifice juice reward to view the faces of high-status males and female perinea (Deaner et al., 2005). When viewing a social scene, humans (Smilek et al., 2006; Birmingham et al., 2008a,b, 2009; Bindemann et al., 2010) and monkeys (McFarland et al., 2013) spend most of the time viewing conspecifics, and faces in particular. In humans, the saliency model fails to account for fixations to faces and saliency values of the locations fixated first are no different than chance (Birmingham et al., 2009).

Our results support these findings, demonstrating that rhesus macaques spend most of their time viewing objects of social relevance when viewing a social scene and that salience does not account for this preference. Furthermore, we found that the three subjects were more correlated in their preference for specific monkeys than objects. Similarly, Deaner, Khera, and Platt found that two males were strongly correlated in their ranked preference for specific faces (Deaner et al., 2005). To understand what social characteristics were most important, we used a model-free, cluster-based approach and found that monkeys that were viewed earlier and longer were more likely to have direct gaze and had redder sex skin, both of which are important visual cues for guiding social behavior (Vandenbergh, 1965; Maestripieri, 1997, 2005; Nunn, 1999; Waitt et al., 2003, 2006; Gerald et al., 2007; Birmingham et al., 2008a; Higham et al., 2013).

It is important to note that further experiments with additional subjects, including females, will be necessary in order to generalize across rhesus monkeys as a group. Another important consideration is that the images used in the present experiment were not photographs of real scenes. However, digitally composed scenes offer far greater control over stimulus features and have been used extensively to study attention and memory (Loftus and Mackworth, 1978; Melcher, 2001; Melcher and Kowler, 2001; Henderson and Hollingworth, 2003; Gajewski and Henderson, 2005; Unema et al., 2005b; Pannasch et al., 2008).

Because this task requires minimal training, allows for the collection of a large amount of data in a short period, and uses stimuli that can be easily altered to manipulate specific factors, it can be used to address a variety of questions about social cognition as well as the neural and hormonal systems regulating it. Oxytocin and vasopressin have long been known to regulate social behavior in rodent species (Ferguson et al., 2000; Young et al., 2001; Donaldson and Young, 2008), but the role of oxytocin in primate social behavior is less well understood (Winslow and Insel, 1991; Boccia et al., 2007; Smith et al., 2010; Chang et al., 2012; Ebitz et al., 2013; Parr et al., 2013; Dal Monte et al., 2014; Simpson et al., 2014).

Because of the importance of maintaining high ecological relevance when studying attention to social stimuli, it will be important going forward to use tasks that elicit social behaviors that are similar to those observed in natural settings (Neisser, 1967; Kingstone et al., 2003; Smilek et al., 2006; Birmingham et al., 2008a,b, 2012; Riby and Hancock, 2008; Bindemann et al., 2009, 2010; Birmingham and Kingstone, 2009). Future experiments using this and other tasks in the rhesus monkey model have the potential to advance our understanding of the neural mechanisms of social behaviors that are disrupted in psychopathologies such as autism spectrum disorder and schizophrenia (Chang and Platt, 2013).

## Author contributions

James A. Solyst and Elizabeth A. Buffalo designed the research, James A. Solyst designed the behavioral task, performed research, and analyzed data, James A. Solyst and Elizabeth A. Buffalo wrote the paper.

### Conflict of interest statement

The authors declare that the research was conducted in the absence of any commercial or financial relationships that could be construed as a potential conflict of interest.
